# Icariside II, a PDE5 Inhibitor, Suppresses Oxygen-Glucose Deprivation/Reperfusion-Induced Primary Hippocampal Neuronal Death Through Activating the PKG/CREB/BDNF/TrkB Signaling Pathway

**DOI:** 10.3389/fphar.2020.00523

**Published:** 2020-04-24

**Authors:** Fan Xu, Chun Lv, Yan Deng, Yuangui Liu, Qihai Gong, Jingshan Shi, Jianmei Gao

**Affiliations:** ^1^Department of Clinical Pharmacotherapeutics, School of Pharmacy, Zunyi Medical University, Zunyi, China; ^2^Department of Pharmacology, Key Laboratory of Basic Pharmacology of Ministry of Education and Joint International Research Laboratory of Ethnomedicine of Ministry of Education, Zunyi Medical University, Zunyi, China

**Keywords:** icariside II, oxygen-glucose deprivation, reoxygenation, primary hippocampal neuron, phosphodiesterase 5, apoptosis

## Abstract

**Background:**

Ischemic stroke remains the leading cause of death and adult disability. Cerebral ischemic/reperfusion (I/R) injury is caused by ischemic stroke thereafter aggravates overwhelming neuronal apoptosis and even the death of neurons. Of note, hippocampus is more susceptive to cerebral I/R injury than the other brain region. This study was designed to explore the effects and mechanism of icariside II (ICS II), a pharmacologically active compound exists in herbal *Epimedii* with previous study-proved as a phosphodiesterase 5 (PDE5) inhibitor, on the oxygen glucose deprivation/reoxygenation (OGD/R)-induced primary hippocampal neurons injury.

**Methods:**

Effects of ICS II on primary hippocampal neuronal impairment and apoptosis induced by OGD/R were examined by MTT, lactate dehydrogenase (LDH) release, TUNEL staining, and flow cytometry, respectively. Activation of memory-related signaling pathways was measured using Western blot analysis. The direct interaction between ICS II and PDE5 was further evaluated by molecular docking.

**Results:**

ICS II (12.5, 25, 50 μM) markedly abrogated OGD/R-induced hippocampal neuronal death as suggested by the increase in neurons viability and the decrease in cellular LDH release. Furthermore, ICS II not only effectively decreased the protein expression and activity of PDE5, restored the 3′5′-cyclic guanosine monophosphate (cGMP) level and its downstream target protein kinase G (PKG) activity but also increased the phosphorylation of cAMP response element binding protein (CREB) level, expressions of brain derived neurotrophic factor (BDNF), and tyrosine protein kinase B (TrkB). Mechanistically, the inhibitory effects of ICS II were abrogated by Rp-8-Br-cGMP (a PKG inhibitor) or ANA-12 (a TrkB inhibitor), which further confirmed that the favorable effects of ICS II were attributed to its activation of the PKG/CREB/BDNF signaling pathways. Intriguingly, ICS II might effectively bind and inhibited PDE5 activity as demonstrated by relatively high binding scores (−6.52 kcal/mol).

**Conclusions:**

ICS II significantly rescues OGD/R-induced hippocampal neuronal injury. The mechanism is, at least partly, due to inhibition of PDE5 and activation of PKG/CREB/BDNF/TrkB signaling pathway. Hence it is thought that ICS II might be a potential naturally PDE5 inhibitor to combat cerebral I/R injury.

## Introduction

Stroke remains one of the dominating cause of death and serious disability around the world (Benjamin et al., 2018). Notably, prevalence of ischemic stroke has overtaken the hemorrhagic one (Feigin et al., 2015). Since ischemic stroke is mostly caused by extracranial embolism or intracranial thrombosis, up to now, thrombolytic therapy is the most robust therapy for ischemic stroke (Saenger and Christenson, 2010). However, cerebral ischemic/reperfusion (I/R) injury caused by restoring blood flow after thrombolytic therapy aggravates neuronal apoptosis and exacerbates neuronal death (Liang et al., 2018). Most importantly, cerebral I/R injury further exacerbates cognitive impairment that is related with neurons apoptosis in hippocampus (Xu et al., 2019), which are more susceptible to cerebral I/R injury than the ones in other brain regions. Unfortunately, so far, effective drugs or strategies to fight cerebral I/R injury are unavailable due to its complex pathogenesis. Therefore, it is imperative to elucidate the mechanism of cerebral I/R injury and develop potent neuroprotective agents.

Emerging evidences show that cerebral I/R injury induces the reduction in brain-derived neurotrophic factor (BDNF) and its receptor tyrosine protein kinase B (TrkB) (Ye et al., 2017; Chen et al., 2018). BDNF phosphorylates TrkB at tyrosine 816 site (Bathina and Das, 2015), once it is phosphorylated exerts multiple neuroprotective effects promoting neuroplasticity, neurogenesis, and angiogenesis (Massa et al., 2010; Schmid et al., 2012), which are related with learning and memory recovery. Moreover, cAMP response element-binding protein (CREB) is a broadly distributed protein located in the nucleus, which can initiate transcription of special genes and then induce growth factor BDNF (Scott Bitner, 2012). Of note, activation of CREB is confirmed to possess anti-apoptosis effect by regulating apoptosis-related proteins in cerebral I/R injury (Zhang W. et al., 2018), suggesting that regulation of CREB/BDNF/TrkB pathway might be a potential strategy in combating cerebral I/R injury.

Phosphodiesterase 5 (PDE5) is one of the PDEs (PDE1-PDE11) family members, which has been demonstrated that exists in human neurons (Teich et al., 2016). PDE5 specifically hydrolyze 3′5′cyclic guanosine monophosphate (cGMP), which is involved in many physiological processes especially in neurons (Wei et al., 2002). Accumulating evidences demonstrate that inhibition of PDE5 can stimulate angiogenesis and neurogenesis after experimental stroke (Ding et al., 2011). These findings indicate that PDE5 inhibitors could probably directly impact on neurons elevating cellular cGMP level and PKG activity, thereby protect against cerebral I/R injury (Chen et al., 2014a). Although classical PDE5 inhibitors, such as sildenafil (SIL), have been proven to have beneficial effects on erectile dysfunction (ED) as well as ischemic stroke, its side effects, including headache, impaired vision, especially, increased risk of melanoma, limited its clinical applications (Loeb et al., 2017). Therefore, it is of great significance to develop novel and safer PDE5 inhibitors. Recently, icariside II (ICS II), a bioactive compound extracted from *Herba Epimedii*, that is used in traditional Chinese medicine (TCM) has been discovered as a novel PDE5 inhibitor (Yin et al., 2016). *Herba Epimedii*, a TCM, has a long history of use in improving sexual performance. It is worth noting that ICS II has also been proven to exhibit salutary effects on ED, as well as neurological disorders (Wang et al., 2015; Yan et al., 2017). Interestingly, although the inhibitory effect of ICS II on PDE5 activity is just half of SIL (Dell'agli et al., 2008), it effectively inhibits melanoma according to recent reports (Wu et al., 2013; Wu et al., 2015). Also encouragingly, our previous studies revealed that ICS II not only ameliorated the neurological dysfunction and decreased infarct volume after middle cerebral artery occlusion-induced cerebral I/R injury in rats (Deng et al., 2016) but also effectively protected against streptozotocin-induced cognitive deficits in rats through decreasing Aβ deposition (Yin et al., 2016). Furthermore, ICS II significantly improved the functions of learning and memory in Alzheimer's disease (AD) mice and upregulated BDNF/TrkB signaling in paralleled *vitro* study (Yan et al., 2017; Liu et al., 2018). Therefore, it is reasonable to assume that ICS II may contribute to restore learning and memory impairments after cerebral I/R insult.

Thus, the present study was designed to explore the effects of ICS II on OGD/R-induced primary hippocampal neurons injury *in vitro* and further to elucidate its underlying mechanism.

## Methods

### Animals

Sprague-Dawley rats were supplied by the Animal Center belonging in the Third Military Medical University. Rats were put in a half day-light/half day-dark cycle, food and water were accessible in the SPF-grade temperature-controlled facilities. All experiments on animal were operated according to the Technology of the People's Republic of China Order No. 2 on November 14, 1988, State Committee of Science and the study protocols were approved by the Experimental Animal Ethics Committee of Zunyi Medical University.

### Reagents

ICS II (HPLC, purity≥98%) was provided by Nanjing Zelang Medical Technology Co., Ltd. (Nanjing, China). ICS II was dissolved in dimethylsulfoxide (DMSO) to 10 mM as the stock solution and diluted in culture medium, and the final concentration of DMSO was less than 0.1%. SIL was purchased from TargetMol (Boston, MA, USA) (T1164). ANA-12 (SML0209), Rp-8-Br-cGMPS sodium salt (SML1614), and MTT (M2128) were supplied by Sigma-Aldric (St Louis, MO, USA). SIL, ANA-12, and Rp-8-Br-cGMP. were dissolved in PBS solution and diluted in medium. Neurobasal-A medium (10888-022) and B27 supplements (17504-044) were purchased from Gibco (Waltham, MA, USA). The Earle's balanced salt solution (EBSS) (top0067) was purchased from Biotopped (Beijing, China). LDH (20180328), PDE5 (20180629), cGMP (20180122), PKG (20180131) ELISA kits were purchased from Shanghai Jiang Lai Biotechnology (Shanghai, China). PDE5 activity kit (GMS50233.3) was brought from GENMED (Shanghai, China). One-step TUNEL assay apoptosis kit (11684817910) was obtained from Roche (Philadelphia, USA). AV/PI apoptosis kit (A005-3) was purchased from Seven Sea biotech (Shanghai, China). Anti-NSE (ab53025), anti-Bax (ab32503), anti-Bcl-2 (ab59348), anti-Caspase-3 (ab13847), anti-BDNF (ab108319), anti-CREB (1:1000, ab32515), anti-phospho-CREB (Ser133) (ab32096), and anti-TrkB (ab18987) were obtained from Abcam (Cambridge, UK). Anti-phospho-TrkB (Tyr816) (4168S) was purchased from Cell Signaling Technology (Shanghai, China). Secondary antibody HRP conjunction AffiniPure goat anti-mouse/rabbit IgG (H+L) (SA00001-1, SA00001-2) were from Proteintech (Rosemont, USA), Alexa Fluor 488 goat anti-rabbit IgG (H+L) (ab150077) was purchased from Abcam (Cambridge, UK).

### Primary Hippocampal Neurons Culture

Primary hippocampal neurons were extracted from newly born rats within 48 h after birth, the dissected hippocampus tissues were sheared separately into small fragments and digested in 0.125% trypsin for 5 min, then added DME/F-12 medium with 10% foetal bovine serum. The mixture was subjected to centrifugal separation at 1000 × for 7 min at 4°C. The neurons were resuspended in DME/F-12 medium, then planted on neuron serum-free cell culture 6-well plates for 4 h. After cells attached, the medium was changed to neurobasal-A medium with 2% B27 supplements. After 8 d, the neurons were washed with PBS, then fixed by 4% paraformaldehyde for 20 min, after that 0.3% Triton X-100 was used to permeabilizated the membranes for another 20 min. The neurons were blocked with 10% goat serum then labeled with neuron-specific enolase (NSE) (1:500), followed by the incubation with fluorescent secondary antibody (1:300) and observed by fluorescence microscope.

### OGD/R Modeling and Treatment

Primary hippocampal neurons were subjected to OGD 1.5 h/R 24 h to mimic cerebral I/R injury *in vitro* as describe in the previous studies (Mo et al., 2016; Feng et al., 2018). In brief, the neurobasal-A culture medium was removed and changed with EBSS (without glucose), subsequently, the cells were put into the hypoxia chamber (STEMCELL) with an atmosphere of 5% CO_2_, 95% N_2_, and 37°C for 1.5 h. After OGD, the medium was transformed into normal culture medium, or different concentrations of ICS II (12.5, 25, 50 μM) or SIL(20 μM) with 5% CO_2_ at 37°C for another 24 h. The specific PKG inhibitor Rp-8-Br-cGMP (30 μM) or TrkB inhibitor ANA-12 (10 μM) was added into the corresponding cells 2 h prior to OGD, respectively.

### Determination of Cell Viability

Cell viability was determined using an MTT assay. In brief, neurons were cultured in 96-well plates at a density of 1 × 10^5^ per well. Then, hippocampal neurons were treated as mentioned above. Following the treatment, MTT solution was added to each well for 4 h incubation at 37°C. After the incubation, the cultured liquid was discarded and replaced with DMSO for dissolving the formazan formed in viable cells. Thereafter, the absorbance value of DMSO solution was measured with microplate reader at 490 nm. Results were represented as the percentage of MTT decrease relative to the control group. For comparison with the effects of ICS II, SIL was used as a positive control for its protective effect against cell death and inhibitory effect of PDE5 activity.

### Determination of Lactate Dehydrogenase Release

Cytotoxicity was measured by detecting the content of lactate dehydrogenase (LDH). Briefly, neurons were treated as mentioned above. The amounts of LDH were measured by ELISA kit at 450 nm. The results of LDH release were shown as optical density LDH level of the medium/(LDH level of medium + cell homogenate LDH level) × 100%.

### Observation of the Morphological Alterations

The hippocampal neurons were treated as described above. The morphological alternation of neurons was pictured using phase contrast microscopy.

### TUNEL Staining

The apoptosis of neurons was detected by one-step TUNEL assay apoptosis kit. In brief, the neurons were treated as mentioned above, then wash with PBS for three times, the neurons were fixed by 4% paraformaldehyde for 20 min, after that 0.3% Triton X-100 was used to permeabilizated the membranes, then added 50 μl TUNEL reaction reagent to each sample, and then incubated for 1 h at 37°C in a humid atmosphere in dark, then cell nucleus were stained by DAPI solution for 5 min, thereafter, the neurons were washed with PBS and visualized by fluorescence microscopy (Olympus IX73; Olympus, Japan) with emission/excitation (515/565 nm) filter. The degree of apoptosis was presented as TUNEL-positive cells (green)/total cells (DAPI).

### Flow Cytometry Analysis

The neuronal apoptosis rates were further determined by flow cytometry analysis. AV/PI apoptosis kit (A005-3; Seven Sea, Shanghai, China) was applied for cell staining. In brief, the neurons were treated as described above. Then, the neurons were resuspended with 400-μl binding solution for each sample, which were stained by annexin-V (AV) and propidium iodide (PI) for 15 min and 5 min in dark, respectively. Thereafter, the samples were read by flow cytometry (ACEA Novocyte, San Diego, USA) and analyzed using NovoExpress software.

### Measurement of PDE5, cGMP, PKG Level, and PDE5 Activity

The PDE5, cGMP, and PKG levels were determined according to ELISA kit, and PDE5 activity was measured by cellular PDE5 activity kit. Briefly, the hippocampal neurons were treated as described above. Thereafter, the neurons were lysed with freezing and freeze-thaw cycles method, then the lysis mixture was centrifuged for 20 min at 12,000×*g*. Cellular PDE5 catalyzed cGMP into 5′-GMP, and further developed into guanosine and free phosphate radical catalyzed by 5-necleotidase, and then phosphate radical was reaction with malachite green. Finally, the results were read by spectrophotometer at 660 nm, and PDE5 activity was represented as the phosphate radical production rate. With existence or inexistence of PDE5 sensitive inhibitor MY-5445, PDE5 activity could be determined.

### Western Blot Analysis

In brief, the hippocampal neurons were treated as described above. Then, the hippocampal neurons were lysed by RIPA buffer and quantified using the BCA assay. Subsequently, samples containing 15 μg protein were electrophoresed and separated on 8% to 12% sodium dodecyl sulfate sodium salt acrylamide gels, and then transferred them onto the membrane of polyvinylidene fluoride. Following blocked with 5% fat-free milk for 1.5 h, the membranes were incubated with the flowing antibodies: anti-Bax (1:1000), anti-Bcl-2 (1:1000), anti-caspase-3 (1:2000), anti-BDNF (1:1000), anti-CREB (1:1000), anti-phospho-CREB (Ser133) (1:500), anti-TrkB (1:1000), and anti–phospho-TrkB (Tyr816) (1:1000) at 4°C overnight. Thereafter, HPR-conjugated antibodies were incubated with specific membrane, respectively. Eventually, electrochemical luminescence reagents kit was used for protein visualizing, and images were quantitative analyzed by Image Lab software (Bio-Rad, Philadelphia, PA, USA).

### Molecular Docking

The interaction between ICS II and PDE5 was performed using Autodock 4.2 and AutodockTools (ADT). The PDE5 was obtained from the X-ray crystal structure of catalytic domain of PDE5 (Protein Data Bank ID: 2H40), then conditioned using ADT by deleting all hydrone and adding all polar hydrogen atoms. A three-dimensional structure of ICS II was established using the ChemBio3D Ultra 14.0 (PerkinElmer Informatics, USA), which was further proceeded using ADT. The area of grid box was included the possible binding sites between ICS II and PDE5 as previous study (Wang et al., 2006).

### Statistical Analysis

All results were presented as mean ± SD and processed by the SPSS 18.0 statistics software. All experiments were performed at least three times. The one-way ANOVA of variance followed by least significant difference *post hoc* test was adopted to determined individual difference. Differences with *P* < 0.05 were considered statistically significant.

## Results

### ICSII Attenuated OGD/R-Induced Injury in Cultured Primary Hippocampal Neurons

**Figure 1:**
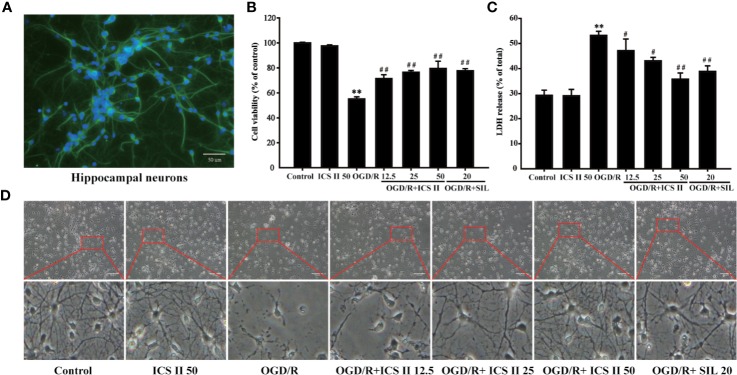
ICS II protected against OGD/R-induced primary hippocampal neurons injury. **(A)** After 8 d cultured, NSE was labeled on hippocampal neurons, further observed by fluorescence microscope. **(B)** Neurons were induced by OGD 1.5 h then reperfusion 24 h, different concentrations of ICS II (12.5, 25, or 50 μM) were administrated during the reperfusion, and the cell viability was determined by MTT assay. **(C)** LDH release from hippocampal neurons was measured using an LDH release assay. **(D)** The protective effects of ICS II on OGD/R-induced morphological alternation. Data are presented as means ± SD (n = 3). ^**^P < 0.01 versus control group; ^#^*P* < 0.05, ^##^*P* < 0.01 versus OGD/R group.

First, NSE was used to label the primary hippocampal neurons for identification. The results showed that the numbers of hippocampal neurons were reached to 92.6 ± 3.2% (**Figure 1A**), which guaranteed the experimental accuracy. Next, we investigated whether ICS II could protect against OGD/R-induced hippocampal neurons injury. The results showed that OGD 1.5 h/R 24 h dramatically decreased cell viability than that of the control group; however, ICS II (12.5, 25, 50 μM) significantly repressed reduction in cell survival in a concentration-dependent manner [F_(6,__14)_=21.369, *P* < 0.001] (**Figure 1B**). In parallel, OGD/R markedly aggravated the leakage of cellular LDH, whereas ICS II concentration-dependently reduced the LDH release after OGD/R insult (F_(6,__14)_ = 187.952, *P* < 0.001) (**Figure 1C**). In addition, OGD/R largely altered the morphologic characteristics, showing the future of dead neurons (neurons detached from the bottom and became shrunken). Whereas, ICS II maintained the integrity of cells and growth rate after OGD/R insult (**Figure 1D**). SIL, a typical PDE5 inhibitor, was selected as positive drug based on numerous studies report that SIL exerts neuroprotective and neurorestorative effects (Chen et al., 2014b). These findings demonstrated that ICS II effectively attenuated OGD/R-induced injury in cultured primary hippocampal neurons, and ICS II at 50 μM exerted better protective effects than SIL.

### ICS II Alleviated OGD/R-Induced Hippocampal Neuronal Apoptosis

**Figure 2:**
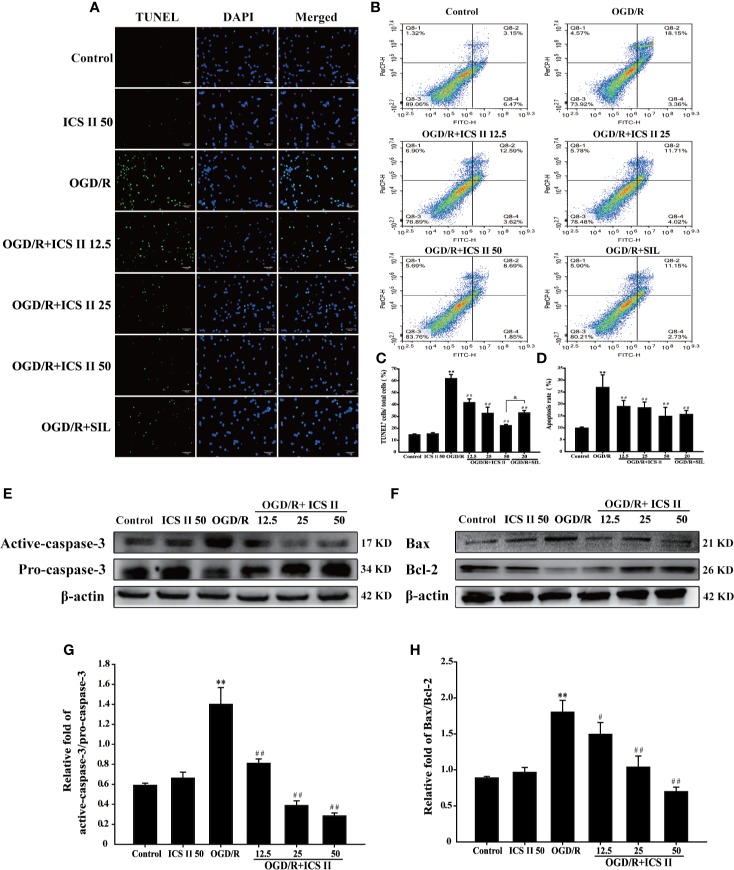
ICS II mitigated OGD/R-induced hippocampal neurons apoptosis. **(A)** Apoptosis cells determined with TUNEL staining (green). **(B)** Neuronal apoptosis rates were determined with AV/PI double staining. **(C)** Statistical results of TUNEL positive neurons/total neurons. **(D)** Statistical results of apoptosis rate at early and late stage. **(E)** Representative Western blot images of cleaved-caspase-3 and caspase-3. **(F)** Representative Western blot images of Bcl-2 and Bax. **(G)** Quantitation of activated-caspase-3/caspase-3. **(H)** Quantitation of Bax/Bcl-2. Data are presented as means ± SD (n = 3). ^**^P < 0.01 versus control group; ^#^*P* < 0.05, ^##^*P* < 0.01 versus OGD/R group; ^&^*P*<0.01 versus OGD/R + ICS II 50 group.

Since neuronal apoptosis is a consequence of cerebral I/R injury (Ko et al., 2009), it is beneficial that if ICS II could alleviate neuronal apoptosis. The results demonstrated that ICS II concentration-dependently lowered the rates of TUNEL positive neurons after OGD/R insult [F_(6,__14)_=106.019, *P* < 0.001] (**Figures 2A, C**). Next, we further investigated which stages of apoptosis were alleviated by ICS II. The results showed that both early and late stage apoptotic cells were decreased by ICS II after OGD/R insult [F_(5,__12)_=10.151, *P* < 0.001] (**Figures 2B–D**). Moreover, SIL also significantly alleviated hippocampal neuronal apoptosis, however, those effects were overtaken by ICS II reflected in TUNEL staining statistically (**Figures 2A, C**). Furthermore, ICS II also significantly reversed the increase of Bax/Bcl-2 ratio, as well as up-regulation of cleaved-caspase-3/caspase-3 than those of OGD/R group [F_(5,__12)_=75.908, *P* < 0.001; F_(5,__12)_=17.654, *P* < 0.001] (**Figures 2E, F**). These findings indicated that the inhibitory effect of ICS II, at least partly, through hindering caspase 3-dependent apoptosis signaling pathway.

### ICS II Enhanced CREB/BDNF/TrkB Pathway After OGD/R

**Figure 3:**
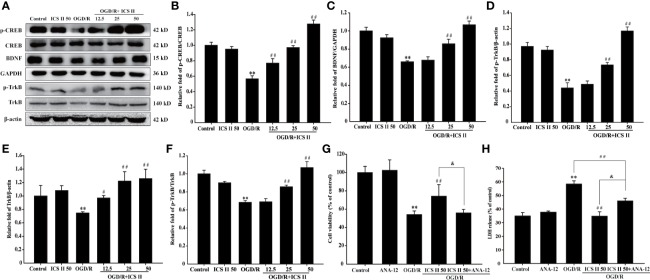
ICS II up-regulated the expressions of BDNF, p-TrkB, TrkB, and p-CREB after OGD/R insult. **(A)** Representative Western blot images of BDNF, p-TrkB, and TrkB. **(B)** Quantitation of BDNF. **(C)** Quantitation of p-TrkB. **(D)** Quantitation of TrkB. **(E)** Representative Western blot images of p-CREB and CREB. **(F)** Quantitation of p-CREB/CREB. TrkB-specific inhibitor ANA-12 abolished the beneficial effects of ICS II. **(G)** Cell viability tested by MTT assay. **(H)** LDH release was detected using an LDH release assay kit. Data are presented as means ± SD (n = 3). ^**^P < 0.01 versus control group; ^#^*P* < 0.05, ^##^*P* < 0.01 versus OGD/R group, ^&^*P* < 0.01 versus OGD/R+ ICS II 50 group.

Next, we investigated the protein expressions of BDNF and TrkB, and the levels of p-TrkB and p-CREB. As we expected, OGD/R markedly decreased the BDNF and TrkB expressions, p-TrkB/TrkB and p-CREB/CREB levels, whereas ICS II increased all those expressions in a concentration-dependent manner [F_(5,__12)_=87.927; F_(5,__12)_=6.835, *P* < 0.001; F_(5,__12)_=127.142, *P* < 0.001] (**Figures 3A–D**). Furthermore, we determined the role of TrkB during the inhibitory effects of ICS II on OGD/R-induced neuronal injury using its specific inhibitor ANA-12. The results showed that ANA-12 partially reversed the protective effects of ICS II, which reflected in results of cell viability and LDH release [F_(4,__10)_=398.576, *P* < 0.001; F_(4,__10)_=102.365, *P* < 0.001] (**Figures 3E, F**). These findings supported the hypothesis that ICS II attenuated hippocampal neuronal apoptosis through activating CREB/BDNF/TrkB pathway.

### ICS II Enhanced cGMP Level and PKG Activity After OGD/R

**Figure 4:**
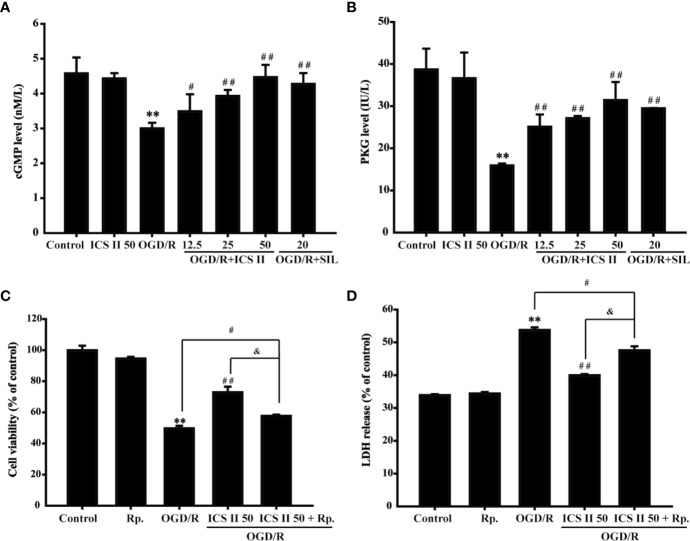
ICS II enhanced cGMP level and PKG activity **(A)** cGMP protein expression was detected by ELISA kit. **(B)** PKG activity was determined by PKG activity kit. PKG-specific inhibitor Rp-8-Br-cGMP (Rp.) abolished the beneficial effects of ICS II. **(C)** Cell viability tested by MTT assay. **(D)** LDH release was detected using an LDH release assay kit. ^**^P < 0.01 versus control group; ^#^*P* < 0.05, ^##^*P* < 0.01 versus OGD/R group, ^&^*P* < 0.01 versus OGD/R+ ICS II 50 group.

cGMP, an important intracellular second messenger, plays an important role in neuron physiology (Wei et al., 2002). It is reported that phosphorylation of CREB could be triggered by cGMP, thus, contributing to memory improvement (Zhang et al., 2018a). The results showed that OGD/R markedly decreased the level of cGMP and PKG activity, whereas ICS II effectively improved cGMP level and PKG activity [F_(6,__14)_=14.956, *P* < 0.001; F_(6,__14)_=26.428, *P*< 0.001] (**Figures 4A, B**). Moreover, to further confirm the role of cGMP in the protective effects of ICS II on hippocampal neuronal injury, cell permeable PKG inhibitor Rp-8-Br-cGMP was used. It was found that Rp-8-Br-cGMP partially reversed the beneficial effect of ICS II, which reflected in result of cell viability and LDH release [F_(4,__10)_=4021.345, *P* < 0.001; F_(4,__10)_=512.811, *P* < 0.001] (**Figures 4C, D**). Collectively, these findings indicated that ICS II protected against OGD/R-induced hippocampal neuronal injury, at least partly, through activating cGMP/PKG pathway.

### ICS II Bound and Inhibited PDE5 After OGD/R Insult

**Figure 5:**
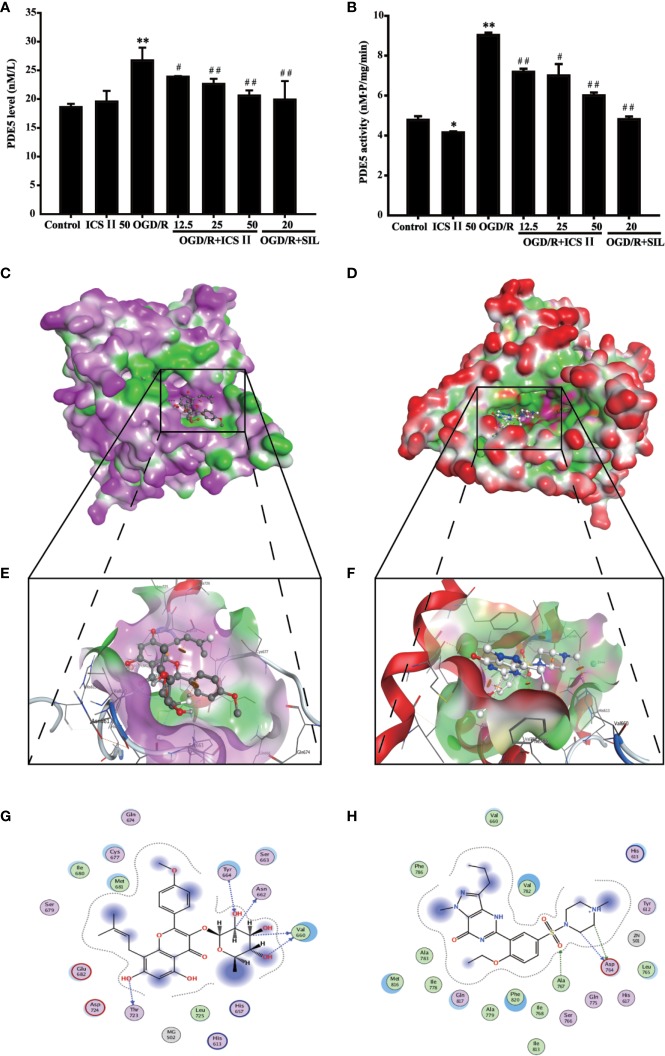
ICS II binds and inhibits PDE5. **(A)** PDE5 protein expression was detected by ELISA kit. **(B)** PDE5 activity was detected by PDE5 activity kit. **(C)** Visual result of binding surface between ICS II and PDE5. **(D)** Visual result of binding sites between ICS II and PDE5. **(E)**. Visual result of binding surface between SIL and PDE5. **(F)** Visual result of binding sites between SIL and PDE5. **(G)** 2D interaction sides between ICS II and PDE5. **(H)** 2D interaction sides between SIL and PDE5. Data are presented as means ± SD (n = 3). ^*^P < 0.05, ^**^P < 0.01 versus control group; ^#^*P* < 0.05, ^##^*P* < 0.01 versus OGD/R group.

The results found that both protein expression and activity of PDE5 were increased after OGD/R, whereas ICS II or SIL effectively lowered PDE5 protein expression and its activity [F _(6,__14)_=236.562, *P* < 0.001; F_(6,__14)_=187.925, *P* < 0.001] (**Figures 5A, B**). Interestingly, virtual molecular docking was adapted to verify whether ICS II or SIL could bind PDE5 protein. The results showed that crystal structure of the catalytic domain of PDE5, a primary functional structure of PDE5 protein, well combined with ICS II or SIL according to their binding energy (−6.52 kcal/mol and −7.8 kcal/mol, respectively). Notably, the results also confirmed that ICS II docked complex has 10 interactional sides with chain and hydrogen binding with the side chain of the ASP724, HIS 675, THR723, and VAL660 residues (**Figure 5G**). Whereas, SIL docked complex has 11 interactional sides with chain (**Figure 5H**), but no hydrogen binding with the side chain (**Figures 5C–H**). Taken together, the findings indicated that ICS II might directly bind with PDE5, thereby exerted neuroprotective effect.

## Discussion

**Figure 6:**
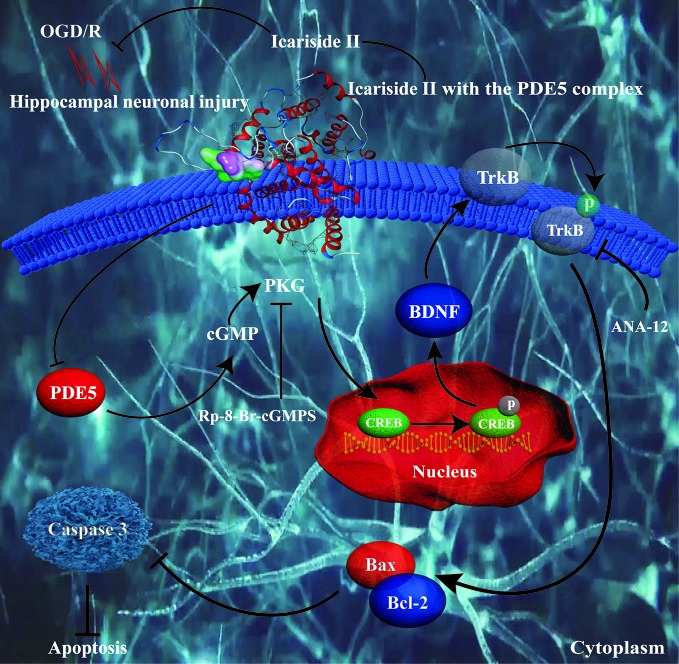
Presentation of a proposed mechanism for the neuroprotective role of ICS II against OGD/R-induced injury in primary hippocampal neurons. ICS II is thought to direct bind and inhibit PDE5, thereby elevating cGMP level and PKG activity. Then, cGMP promoted PKG/BDNF/TrkB/CREB pathways, thereby reducing neuronal apoptosis and assisting neuronal survival.

The present study revealed that: (1) ICS II protected against OGD/R-induced primary hippocampal neuronal impairments. (2) PKG/CREB/BDNF/TrkB signaling was correlated the neuroprotective effects of ICS II on hippocampal neurons. (3) Most importantly, ICS II inhibited PDE5, and its inhibitory effect was possibly associated with direct interaction between them (**Figure 6**).

During cerebral I/R injury, neuronal apoptosis happened in hippocampus can lead to cognitive impairments and other neurological deficits (Yang et al., 2017; Shang et al., 2018). Therefore, protection of hippocampus against cerebral I/R injury is becoming a crucial strategy in drug treatment. Ischemic stroke blocks the nutritional supplies of neurons, especially oxygen and glucose, which are the most important supplies of neurons. Therefore, OGD/R-induced neuronal injury is usually used as an *in vitro* model to mimic cerebral I/R injury (Tang et al., 2017). In the present study, our findings demonstrated ICS II reduced primary hippocampal neurons injury after OGD/R insult, as demonstrated by increased in cell viability, reduced in LDH release and neuronal apoptosis. These data suggested that ICS II against cerebral I/R injury, and thus, it might be contributed to ameliorate neurological deficits and cognitive impairments.

As a part of neurotrophic family of proteins, BDNF is extensively distributes in mammalian central nervous system and is involved in normal brain development and in cognitive processes (Park and Poo, 2013). It has been demonstrated that BDNF is strongly beneficial to the survival of neurons *via* activating anti-apoptotic pathways (Jantas et al., 2009; Zhang et al., 2011). Notably, the present study demonstrated that OGD/R inactivated the phosphorylation of CREB, thereby decreased BDNF and TrkB expressions, as well as p-TrkB level. However, ICS II effectively reversed these changes, which suggested that CREB/BDNF/TrkB pathway was involved in the beneficial effect of ICS II on cerebral I/R injury. Intriguingly, the TrkB inhibitor ANA-12 almost abolished the inhibition effects of ICS II on hippocampal neuronal injury, which further confirmed that the CREB/BDNF/TrkB pathway exerted a direct role in the salutary effects of ICS II on hippocampal neuronal apoptosis after OGD/R insult. Furthermore, several studies have shown that activation of BDNF/TrkB was involved in inhibition of Bax/Bcl-2 ratio, a typical apoptotic index, after cerebral I/R injury (Zhao et al., 2018). Moreover, inhibition of phosphorylation of TrkB increases Bax/Bcl-2 ratio will further activate caspase-3 (Zhao et al., 2018). Our findings elucidated that ICS II markedly suppressed the enhancement of Bax/Bcl-2 ratio and activation of caspase-3 after OGD/R insult, suggesting that ICS II attenuated cerebral I/R injury, at least partly, through activating CREB/BDNF/TrkB signaling pathway and caspase 3-dependant apoptosis pathway.

Notably, PDE5 not only plays an imperative role in learning and memory function *via* regulating the cGMP/PKG signal pathways, thereby activating phosphorylation of CREB as previous reports have shown (Zhang et al., 2018b) but also plays a crucial role in cerebral I/R injury as evidenced by our previous study (Deng et al., 2016). In addition, PKG could induce BDNF transcription *via* CREB in the nucleus, and activate it downstream TrkB (Lu et al., 1999). Thus, targeting PDE5 is a promising strategy for treating neurological diseases, such as stroke and AD. Intriguingly, this study showed that ICS II significantly attenuated the expression and activity of PDE, while it increased cGMP level and PKG activity, consisting with our previous study (Gao et al., 2017; Gao et al., 2018). Furthermore, the favorable effects of ICS II were almost abolished by Rp-8-Br-cGMP, a PKG inhibitor, which further confirmed that ICS II protected against OGD/R-induced neuronal injury through regulating cGMP-dependent pathway. Although this study and our previous studies manifested that ICS II effectively inhibited PDE5 expression and its activity, whether ICS II targeting PDE5 could inhibit cerebral I/R-induced injury in hippocampal neurons remains still unclear. Interestingly, by the result of molecular docking, we confirmed that ICS II directly combinate with the catalytic domain of PDE5, which provided a robust theoretical foundation for that ICS II is a PDE5 inhibitor. Additionally, the results also demonstrated that SIL showed higher affinity with PDE5 than ICS II, which could explain why SIL exerts better inhibitory effect of PDE5 than that of ICS II, consisting with previous study (Dell'agli et al., 2008).

Of note, the present study was focused on the preliminary neuroprotective effect of ICS II on OGD/R-induced hippocampal neurons injury *in vitro*. The beneficial effects of ICS II on cerebral I/R-induced learning and memory impairment *in vivo*, and the detailed mechanisms of reciprocity between CREB/BDNF/TrkB and cGMP/PKG will be further elucidated in our next story.

## Conclusion

In summary, this study reveals that ICS II exerts beneficial effects on OGD/R-induced hippocampal neuronal apoptosis, at least partially, though mediation of PDE5/cGMP/PKG axis and CREB/BDNF/TrkB signaling pathway. These findings raise the possibility that ICS II may be developed into a therapeutic agent against cerebral I/R-induced cognitive impairments.

## Data Availability Statement

The original contributions presented in the study are included in the article/supplementary files, further inquiries can be directed to the corresponding authors.

## Ethics Statement

Animal experiments were performed according the State Committee of Science and Technology of the People's Republic of China Order No. 2 on November 14, 1988, and the study protocol was approved by the Experimental Animal Ethics Committee of the Zunyi Medical University.

## Author Contributions

JG, QG and JS designed the experimental approaches. FX performed all the other studies described herein, except the Western blot experiments conducted by CL and YD, Annexin V-FITC/PI staining by YL. FX wrote the manuscript with the help from QG and JG.

## Funding

This work was supported by Natural Science Foundation of China (Grant No. 81760727), Program for excellent young talents of Zunyi Medical University (Grant No. 15zy-002), Science and Technology Innovation Talent Team of Guizhou Province (Grant No.20154023), the Education Department of Guizhou Province of China (Grant No. GNYL (2017) 006, YLXKJS-YS-06), the Education Department of Guizhou Province of China (Grant No. GNYL (2017) 006, YLXKJS-YS-06) and Program for Changjiang Scholars and Innovative Research Team in University, China (Grant No. IRT_17R113).

## Conflict of Interest

The authors declare that the research was conducted in the absence of any commercial or financial relationships that could be construed as a potential conflict of interest.

## References

[B1] Bathina S. Das U. N. (2015). Brain-derived neurotrophic factor and its clinical implications. Arch. Med. Sci. 11, 1164–1178. 10.5114/aoms.2015.56342 26788077 PMC4697050

[B2] Benjamin E. J. Virani S. S. Callaway C. W. Chamberlain A. M. Chang A. R. Cheng S. (2018). Heart Disease and Stroke Statistics-2018 Update: A Report From the American Heart Association. Circulation 137, e67–e492. 10.1161/CIR.0000000000000558 29386200

[B3] Chen X. Wang N. Liu Y. Liu Y. Zhang T. Zhu L. (2014a). Yonkenafil: a novel phosphodiesterase type 5 inhibitor induces neuronal network potentiation by a cGMP-dependent Nogo-R axis in acute experimental stroke. Exp. Neurol. 261, 267–277. 10.1016/j.expneurol.2014.07.007 25064698

[B4] Chen X. M. Wang N. N. Zhang T. Y. Wang F. Wu C. F. Yang J. Y. (2014b). Neuroprotection by sildenafil: neuronal networks potentiation in acute experimental stroke. CNS Neurosci. Ther. 20, 40–49. 10.1111/cns.12162 24034153 PMC6493202

[B5] Chen C. M. Wu C. T. Yang T. H. Liu S. H. Yang F. Y. (2018). Preventive Effect of Low Intensity Pulsed Ultrasound against Experimental Cerebral Ischemia/Reperfusion Injury via Apoptosis Reduction and Brain-derived Neurotrophic Factor Induction. Sci. Rep. 8, 5568. 10.1038/s41598-018-23929-8 29615782 PMC5882812

[B6] Dell'agli M. Galli G. V. Dal Cero E. Belluti F. Matera R. Zironi E. (2008). Potent inhibition of human phosphodiesterase-5 by icariin derivatives. J. Nat. Prod. 71, 1513–1517. 10.1021/np800049y 18778098

[B7] Deng Y. Xiong D. Yin C. Liu B. Shi J. Gong Q. (2016). Icariside II protects against cerebral ischemia-reperfusion injury in rats via nuclear factor-kappaB inhibition and peroxisome proliferator-activated receptor up-regulation. Neurochem. Int. 96, 56–61. 10.1016/j.neuint.2016.02.015 26939761

[B8] Ding G. Jiang Q. Li L. Zhang L. Zhang Z. Lu M. (2011). Longitudinal magnetic resonance imaging of sildenafil treatment of embolic stroke in aged rats. Stroke 42, 3537–3541. 10.1161/STROKEAHA.111.622092 21903952 PMC3226838

[B9] Feigin V. L. Krishnamurthi R. V. Parmar P. Norrving B. Mensah G. A. Bennett D. A. (2015). Update on the Global Burden of Ischemic and Hemorrhagic Stroke in 1990-2013: The GBD 2013 Study. Neuroepidemiology 45, 161–176. 10.1159/000441085 26505981 PMC4633282

[B10] Feng L. Gao J. Liu Y. Shi J. Gong Q. (2018). Icariside II alleviates oxygen-glucose deprivation and reoxygenation-induced PC12 cell oxidative injury by activating Nrf2/SIRT3 signaling pathway. Biomed. Pharmacother. 103, 9–17. 10.1016/j.biopha.2018.04.005 29635133

[B11] Gao J. Deng Y. Yin C. Liu Y. Zhang W. Shi J. (2017). Icariside II, a novel phosphodiesterase 5 inhibitor, protects against H2 O2 -induced PC12 cells death by inhibiting mitochondria-mediated autophagy. J. Cell Mol. Med. 21, 375–386. 10.1111/jcmm.12971 27642051 PMC5264130

[B12] Gao J. Xu Y. Lei M. Shi J. Gong Q. (2018). Icariside II, a PDE5 inhibitor from Epimedium brevicornum, promotes neuron-like pheochromocytoma PC12 cell proliferation via activating NO/cGMP/PKG pathway. Neurochem. Int. 112, 18–26. 10.1016/j.neuint.2017.10.015 29101001

[B13] Jantas D. Szymanska M. Budziszewska B. Lason W. (2009). An involvement of BDNF and PI3-K/Akt in the anti-apoptotic effect of memantine on staurosporine-evoked cell death in primary cortical neurons. Apoptosis 14, 900–912. 10.1007/s10495-009-0370-6 19521778

[B14] Ko I. G. Shin M. S. Kim B. K. Kim S. E. Sung Y. H. Kim T. S. (2009). Tadalafil improves short-term memory by suppressing ischemia-induced apoptosis of hippocampal neuronal cells in gerbils. Pharmacol. Biochem. Behav. 91 (4), 629–635. 10.1016/j.pbb.2008.10.009 19010346

[B15] Liang Y. Xu J. Wang Y. Tang J. Y. Yang S. L. Xiang H. G. (2018). Inhibition of MiRNA-125b Decreases Cerebral Ischemia/Reperfusion Injury by Targeting CK2alpha/NADPH Oxidase Signaling. Cell Physiol. Biochem. 45, 1818–1826. 10.1159/000487873 29510389

[B16] Liu S. Li X. Gao J. Liu Y. Shi J. Gong Q. (2018). Icariside II, a Phosphodiesterase-5 Inhibitor, Attenuates Beta-Amyloid-Induced Cognitive Deficits via BDNF/TrkB/CREB Signaling. Cell Physiol. Biochem. 49, 985. 10.1159/000493232 30196289

[B17] Loeb S. Ventimiglia E. Salonia A. Folkvaljon Y. Stattin P. (2017). Meta-Analysis of the Association Between Phosphodiesterase Inhibitors (PDE5Is) and Risk of Melanoma. J. Natl. Cancer Inst. 109 (8), djx086. 10.1093/jnci/djx086 PMC5437700 29117385

[B18] Lu Y. F. Kandel E. R. Hawkins R. D. (1999). Nitric oxide signaling contributes to late-phase LTP and CREB phosphorylation in the hippocampus. J. Neurosci. 19, 10250–10261. 10.1523/JNEUROSCI.19-23-10250.1999 10575022 PMC6782403

[B19] Massa S. M. Yang T. Xie Y. Shi J. Bilgen M. Joyce J. N. (2010). Small molecule BDNF mimetics activate TrkB signaling and prevent neuronal degeneration in rodents. J. Clin. Invest. 120, 1774–1785. 10.1172/JCI41356 20407211 PMC2860903

[B20] Mo Z. T. Li W. N. Zhai Y. R. Gong Q. H. (2016). Icariin Attenuates OGD/R-Induced Autophagy via Bcl-2-Dependent Cross Talk between Apoptosis and Autophagy in PC12 Cells. Evid. Based Complement Alternat. Med. 2016, 4343084. 10.1155/2016/4343084 27610184 PMC5004044

[B21] Park H. Poo M. M. (2013). Neurotrophin regulation of neural circuit development and function. Nat. Rev. Neurosci. 14, 7–23. 10.1038/nrn3379 23254191

[B22] Saenger A. K. Christenson R. H. (2010). Stroke biomarkers: progress and challenges for diagnosis, prognosis, differentiation, and treatment. Clin. Chem. 56, 21–33. 10.1373/clinchem.2009.133801 19926776

[B23] Schmid D. A. Yang T. Ogier M. Adams I. Mirakhur Y. Wang Q. (2012). A TrkB small molecule partial agonist rescues TrkB phosphorylation deficits and improves respiratory function in a mouse model of Rett syndrome. J. Neurosci. 32, 1803–1810. 10.1523/JNEUROSCI.0865-11.2012 22302819 PMC3710112

[B24] Scott Bitner R. (2012). Cyclic AMP response element-binding protein (CREB) phosphorylation: a mechanistic marker in the development of memory enhancing Alzheimer's disease therapeutics. Biochem. Pharmacol. 83, 705–714. 10.1016/j.bcp.2011.11.009 22119240

[B25] Shang J. L. Cheng Q. Duan S. J. Li L. Jia L. Y. (2018). Cognitive improvement following ischemia/reperfusion injury induced by voluntary runningwheel exercise is associated with LncMALAT1mediated apoptosis inhibition. Int. J. Mol. Med. 41, 2715–2723. 10.3892/ijmm.2018.3484 29436629 PMC5846661

[B26] Tang F. Guo S. Liao H. Yu P. Wang L. Song X. (2017). Resveratrol Enhances Neurite Outgrowth and Synaptogenesis Via Sonic Hedgehog Signaling Following Oxygen-Glucose Deprivation/Reoxygenation Injury. Cell Physiol. Biochem. 43, 852–869. 10.1159/000481611 28957797

[B27] Teich A. F. Sakurai M. Patel M. Holman C. Saeed F. Fiorito J. (2016). PDE5 Exists in Human Neurons and is a Viable Therapeutic Target for Neurologic Disease. J. Alzheimers Dis. 52, 295–302. 10.3233/JAD-151104 26967220 PMC4927884

[B28] Wang H. Liu Y. Huai Q. Cai J. Zoraghi R. Francis S. H. (2006). Multiple conformations of phosphodiesterase-5: implications for enzyme function and drug development. J. Biol. Chem. 281, 21469–21479. 10.1074/jbc.M512527200 16735511

[B29] Wang L. Xu Y. Li H. Lei H. Guan R. Gao Z. (2015). Antioxidant icariside II combined with insulin restores erectile function in streptozotocin-induced type 1 diabetic rats. J. Cell Mol. Med. 19, 960–969. 10.1111/jcmm.12480 25781208 PMC4420599

[B30] Wei J. Y. Jin X. Cohen E. D. Daw N. W. Barnstable C. J. (2002). cGMP-induced presynaptic depression and postsynaptic facilitation at glutamatergic synapses in visual cortex. Brain Res. 927, 42–54. 10.1016/S00006-8993(01)03323-6 11814431

[B31] Wu J. Xu J. Eksioglu E. A. Chen X. Zhou J. Fortenbery N. (2013). Icariside II induces apoptosis of melanoma cells through the downregulation of survival pathways. Nutr. Cancer. 65, 110–117. 10.1080/01635581.2013.741745 23368920

[B32] Wu J. Song T. Liu S. Li X. Li G. Xu J. (2015). Icariside II inhibits cell proliferation and induces cell cycle arrest through the ROS-p38-p53 signaling pathway in A375 human melanoma cells. Mol. Med. Rep. 11, 410–416. 10.3892/mmr.2014.2701 25333296

[B33] Xu F. Zhang G. Yin J. Zhang Q. Ge M. Y. Peng L. (2019). Fluoxetine mitigating late-stage cognition and neurobehavior impairment induced by cerebral ischemia reperfusion injury through inhibiting ERS- mediated neurons apoptosis in the hippocampus. Behav. Brain Res. 370, 111952. 10.1016/j.bbr.2019.111952 31103751

[B34] Yan L. Deng Y. Gao J. Liu Y. Li F. Shi J. (2017). Icariside II Effectively Reduces Spatial Learning and Memory Impairments in Alzheimer's Disease Model Mice Targeting Beta-Amyloid Production. Front. Pharmacol. 8, 106. 10.3389/fphar.2017.00106 28337142 PMC5340752

[B35] Yang R. Hu K. Chen J. Zhu S. Li L. Lu H. (2017). Necrostatin-1 protects hippocampal neurons against ischemia/reperfusion injury via the RIP3/DAXX signaling pathway in rats. Neurosci. Lett. 651, 207–215. 10.1016/j.neulet.2017.05.016 28501693

[B36] Ye X. Yu L. Zuo D. Zhang L. Zu J. Hu J. (2017). Activated mGluR5 protects BV2 cells against OGD/R induced cytotoxicity by modulating BDNF-TrkB pathway. Neurosci. Lett. 654, 70–79. 10.1016/j.neulet.2017.06.029 28642149

[B37] Yin C. Deng Y. Gao J. Li X. Liu Y. Gong Q. (2016). Icariside II, a novel phosphodiesterase-5 inhibitor, attenuates streptozotocin-induced cognitive deficits in rats. Neuroscience 328, 69–79. 10.1016/j.neuroscience.2016.04.022 27109920

[B38] Zhang J. Yu Z. Yu Z. Yang Z. Zhao H. Liu L. (2011). rAAV-mediated delivery of brain-derived neurotrophic factor promotes neurite outgrowth and protects neurodegeneration in focal ischemic model. Int. J. Clin. Exp. Pathol. 4, 496–504. 10.3109/14992027.2010.526637 21738820 PMC3127070

[B39] Zhang L. Seo J. H. Li H. Nam G. Yang H. O. (2018a). The phosphodiesterase 5 inhibitor, KJH-1002, reverses a mouse model of amnesia by activating a cGMP/cAMP response element binding protein pathway and decreasing oxidative damage. Br. J. Pharmacol. 175 (16), 3347–3360. 10.1111/bph.14377 29847860 PMC6057906

[B40] Zhang W. Song J. K. Yan R. Li L. Xiao Z. Y. Zhou W. X. (2018). Diterpene ginkgolides protect against cerebral ischemia/reperfusion damage in rats by activating Nrf2 and CREB through PI3K/Akt signaling. Acta Pharmacol. Sin. 39, 1259–1272. 10.1038/aps.2017.149 29542683 PMC6289361

[B41] Zhang L. Seo J. H. Li H. Nam G. Yang H. O. (2018b). The phosphodiesterase 5 inhibitor, KJH-1002, reverses a mouse model of amnesia by activating a cGMP/cAMP response element binding protein pathway and decreasing oxidative damage. Br. J. Pharmacol. 175, 3347–3360. 10.1111/bph.14377 29847860 PMC6057906

[B42] Zhao Y. Wang J. Du J. Li B. Gou X. Liu J. (2018). TAT-Ngn2 Enhances Cognitive Function Recovery and Regulates Caspase-Dependent and Mitochondrial Apoptotic Pathways After Experimental Stroke. Front. Cell Neurosci. 12, 475. 10.3389/fncel.2018.00475 30618628 PMC6302814

